# PatchCLIP enables region specific contrastive health record and image joint training with patch embedding loss

**DOI:** 10.1038/s41598-026-52235-x

**Published:** 2026-05-09

**Authors:** Sheethal Bhat, Awais Mansoor, Bogdan Georgescu, Mathias Zinnen, Pranjal Sahu, Adarsh B. Panambur, Florin C. Ghesu, Sasa Grbic, Andreas Maier

**Affiliations:** 1 Pattern Recognition Lab, Friedrich-Alexander-Universität, Erlangen-Nürnberg, 91058 Erlangen, Germany; 2 Digital Technology and Innovation, Siemens Healthineers, 90587 Erlangen, Germany; 3 Digital Technology and Innovation, Siemens Medical Solutions, Princeton, NJ 08540 USA

**Keywords:** Computer-assisted diagnosis, CLIP, Vision-language models, Biomedical engineering, Computer science

## Abstract

Vision-Language (VL) models such as Contrastive Language-Image pretraining (CLIP) have shown remarkable zero-shot classification capabilities by jointly learning from large-scale image–text datasets using multimodal self-supervised learning (SSL). However, while these models capture strong global semantics, they often struggle with fine-grained spatial understanding, thereby limiting their effectiveness in downstream tasks like object detection and medical abnormality localization^2^. To address this limitation, we propose Patch-CLIP, a novel VL framework that introduces a contrastive loss aligning image patch-level embeddings with text embeddings. Unlike conventional VL approaches that only leverage global image representations, our method utilizes local patch-level features to encode spatial context, enabling effective learning of localization cues. Applied to two Chest X-ray (CXR) datasets, Patch-CLIP achieves state-of-the-art (SOTA) performance across eight abnormality detection tasks. Furthermore, the resulting patch prediction maps substantially reduce false positives at comparable sensitivity levels compared to standard saliency-based methods, providing more precise and interpretable localization of key findings. The code is available at https://github.com/Siemens-Healthineers/patch-clip

## Introduction

Contrastive Vision-Language (VL) models^2^ address one of the major drawbacks of regular vision models in the medical imaging domain: the labor-intensive and costly nature of the annotation process required to create accurate gold standard annotations for training such models^3–5^. VL models are generally trained over a diverse set of very large amount of data^2,6^. The relation between text and image data is used as a supervision signal to create a proxy training task. This training paradigm is particularly well suited for the medical imaging domain for two main reasons: (1) VL pairing naturally occurs for medical images in the form of clinical reports accompanying the imaging data^7^, and also (2) contrary to the natural imaging domain, where large-scale annotations are often crowd-sourced due to the minimal expertise required, annotation of medical imaging data requires expert(s) (such as a chest radiologist(s) with several years of experience)^3,8^.

Pairing the visual features to the text in an unsupervised manner circumvents the rising cost of expensive medical annotations, time as well as the need for skilled annotators. This is especially true in applications involving image-level classification. However, besides holistic image-level classification performance where VL models have demonstrated reasonable utility^6,9,10^, there is also a growing need for computer-aided diagnostic (CAD) systems to provide auxiliary support to radiologists by facilitating localization^11^. Such systems enable speedier diagnosis and improve clinical workflows through assisting clinicians in assessment and risk estimation^12^.

The localization tasks are notably challenging because radiologists depend on multiple contextual cues to diagnose various pathological findings or abnormalities^13^. These contextual cues and priors imaging exams are typically included in clinical reports, which are underutilized by current state-of-the-art (SOTA) vision based medical abnormality detection systems. Every radiological abnormality in medical domain represents a range of complexities (e.g., comorbidities and mimickers) and priors that a visual AI system trained on anonymized patient data cannot fathom or may not have access to. Therefore, current SOTA abnormality detectors achieve higher accuracy and overlap of region predictions through a large number of training annotations^3,5,14,15^, which often translate to higher cost^4^. Moreover, to prevent annotation bias, comprehensive instructions are necessary to standardize annotations among clinical experts^16,17^, further leading to increased cost.

It is, therefore, anticipated that by utilizing contextual information from clinical reports, the necessity for extensive annotations could potentially be reduced significantly if not completely eliminated^18^. Previous efforts to train an abnormality detection systems solely using clinical reports have largely been either unsuccessful or very limited in scope due to inter-radiologist variability in description within the text reports that rely heavily on previously known yet not captured in the report clinical context and site and personality specific conventions^4,19^. Furthermore, clinical reports generally describe the location approximately rather than precise localizations through tight bounding boxes that are generally needed to train most vision models thus necessitating refinement with precise region-based annotations^3,14,15^.

However, in clinical settings, the accuracy in predicting the correct image region is prioritized over achieving a high overlap between ground truth and predictions, thus motivating the development of systems that perform “coarse” localization^5,8^. This motivates the development of a localization system that can utilize VL based unsupervised models to provide coarse localization with a minimum amount of annotated data.

In this work, we introduce an innovative finetuning method that leverages the fusion of image and corresponding text embeddings that is inspired by CLIP^2^. The CLIP^2^ model correlates the VL pairs globally in the last stage of the network, and maximizes the cosine similarity of these pairs thus minimizing the contrastive loss in the shared latent space. To facilitate localization, we introduce Patch-CLIP with two additional loss functions: a *linear combination loss* and a *patchwise contrastive loss*. The former ensures that the combination of patch-level embeddings is correlated to the text information while the latter ensures the local alignment of patch embeddings with text embeddings. Additionally, we improve feature extraction for the region-of-interest by introducing an additional down-scaler network preceding the image encoder.

**Fig. 1:**
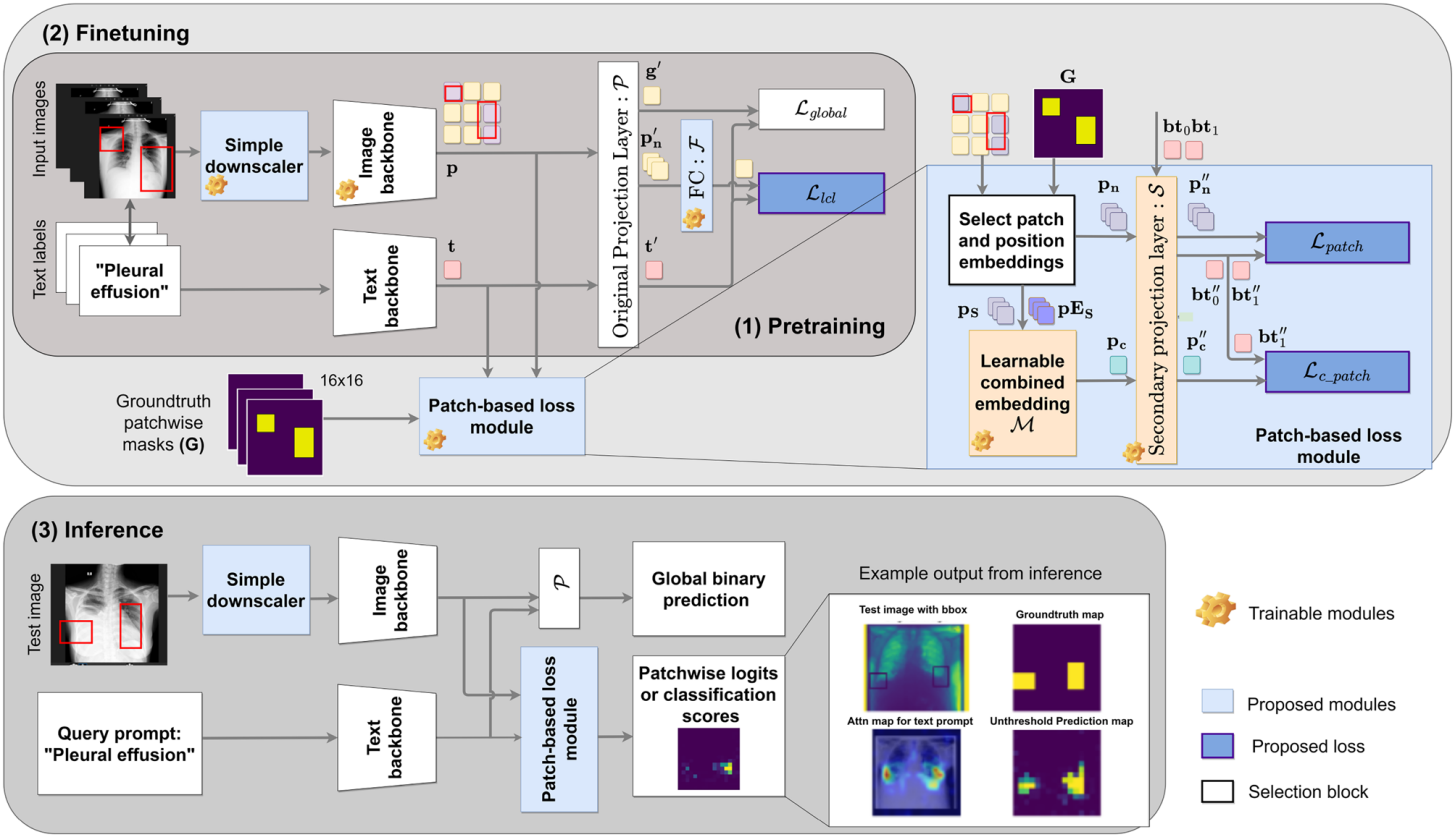
Overview of the finetuning process with the patch embedding loss function and inference method (changes in blue). $L_{lcl}$ is the linear combination loss that amplifies the learning from the patch embeddings and $L_{patch}$ and $L_{c\_patch}$ indicate the patch-wise CE losses from correlating the patch embeddings to text. For inference, a text query prompt is contrasted with the patch embeddings, to generate patch-wise classification probability scores in addition to the original classification scores.

Figure 1 shows the overview of our methodology with our contributions highlighted in color. The novel loss functions are implemented on the extracted patch embedding outputs of the image encoder after their transformation through a secondary projection layer that is depicted in the “Patch-based Loss Module”. Offline-computed ground truth masks from available annotations are used to extract the relevant patch embeddings. During inference, Patch-CLIP is prompted with a text input that represents the finding. Subsequently, the network produces patchwise logits, signifying the probability of the queried finding’s presence. The resulting low-resolution prediction map provides a discretized indication of the location and the inference stage does not need any further evaluation of the indicated locations of the findings.

We evaluated Patch-CLIP on two Chest X-ray (CXR) datasets containing various pulmonary and cardiothoracic abnormalities. CXR exams are uniquely suited for this evaluation due to the difficulty in obtaining reliable annotations and the availability of large-scale public datasets such as MIMIC^20^ derived from radiographic text reports.

Current Large Language Models (LLMs) can parse these existing text reports efficiently by paying attention to key findings which can be used to establish a correlation to the CXR test images.

### Contribution

- We introduce Patch-CLIP, a novel multi-loss framework that leverages a Vision–Language (VL) pretrained classifier to perform abnormality localization and classification simultaneously, without requiring a trade-off between these objectives. The method combines patch contrastive losses with the CLIP loss, enforcing both local (patch-level) and global (semantic) alignment. Patch-CLIP learns adaptive spatial weighting consistent with text semantics, enabling robust weakly supervised localization.
- The Patch-CLIP architecture remains lightweight and efficient, requiring only a single fully connected layer on top of a frozen VL backbone—avoiding transformer decoders or region proposal heads. This simplicity allows training and inference at lower computational cost while maintaining good localization performance under high class imbalance and limited annotation conditions.
- We quantitatively evaluate this method through Free-response Receiver Operating Characteristic (FROC) curves, comparing them to the performance of the saliency maps from the classification model and a SOTA object detection model demonstrating improved sensitivity performance at clinically relevant false positive rates. The method is further evaluated using classification AUC scores against multiple SOTA abnormality detection systems and VL models on two CXR datasets: more than eight findings in the VinDR-CXR dataset^3^ and two findings in an internal CXR dataset^8^.
- We further introduce a downscaler network and pretraining strategy based on the linear combination loss, enabling large-scale pretraining on expanded CXR image–text data. This improves both classification and downstream localization performance. Finally, several ablation studies are performed to analyze the contribution of each component, which contributes to improved performance, data efficiency, and stability under class imbalance.

The paper is organized as follows: first, related works are discussed. This is followed by a detailed explanation of the proposed methodology. The preparation of the data and the experimental setup is then described and the results are presented. Finally, a discussion on the method and results are presented followed by conclusions of the study.

## Related works

Traditional detection and localization approaches can be broadly classified into two categories: those utilizing attention maps to “ground” Deep Learning (DL) predictions^10,21–23^, and those employing an object detection-based pipeline as a downstream training objective^24,25^. These methods can be pure vision-based or VL based. In the context of medical imaging, current evidence suggests that specialized abnormality detection models remain SOTA when compared to generic VL object detection frameworks^5,15,26,27^. In the following subsections, we first review VL classification methods that demonstrate localization through their saliency maps, followed by general VL detection methods that perform well in the natural image space, and finally dedicated vision based detection methods that are current SOTA in medical domain.

### Saliency maps of VL classification methods

Self-supervised (SSL) classification methods in the CXR domains have been extensively researched, with efforts made to enhance the location predictions for various findings^1^. The accuracy of attention or saliency maps in SSL systems is dependent on the method used for pretraining. Vision-Language (VL) methods such as CLIP^2^, ChexZero^6^, and VLCE^28^ correlate the image-text pairs and rely on the attention mechanism to demonstrate localization (which is implicitly learned during pretraining). The method demonstrates superior zero-shot performance as compared to other large-scale unsupervised contrastive learning techniques such as MoCo^29^, SwAV^5^ or DINO^1^, which rely on extracting embeddings learned from contrasting positive and negative samples or clustering the embeddings in latent space.

As the VL pretraining paradigm in CLIP^2^ does not include an image-to-image contrastive loss and only relies on the correlation to the text information, the resulting saliency maps and finetuned performance may not necessarily beat DINO^1^. In CLIP based methods, the attention maps tend to produce a high number of false positives (FP) in the form of spurious activations and fail to guarantee high Intersection-Over-Union (IoU) values^1,2,22^. These methods also process images at much lower resolution typically around 256$\times$256. CXR images are typically high resolution at 2544$\times$3056 and a uniform downsizing of these images may lead to the removal of smaller findings or the modification of crucial identifying information^4,13,30–32^. A study that attempts to include the image-image SimCLR contrastive learning objective along with the VL learning is SLIP^33^. While this pretraining approach enhances the top-5 accuracy on ImageNet^34^, its capability for transfer learning to the medical domain remains limited.

Regarding attention mechanisms, GLORIA^10^ introduces a “multimodal global-local representation learning framework for label efficient recognition”, demonstrating high performance in classification and segmentation on different datasets. The authors contrast image subregions and words in clinical reports to develop an attention-based framework that learns global and local feature representations. On the other hand, BioViL^35^ presents that “principled textual semantic modeling” can substantially improve contrastive learning in SSL visual language processing. The authors introduce a language model that achieves SOTA results in radiology natural inference that is achieved through an increase in vocabulary, novel language pretraining, and by leveraging semantics characteristics in radiology reports.

More recently Wu, et al.^9^ automatically annotated CXRs based on information extracted from radiographic reports with in-depth analysis on various datasets. They accomplished this through the use of a nodule classification model with a PSP-Net and ResNet-50 architecture to achieve SOTA zero-shot classification results. In the same vein, improvements in medical VL pretraining have also been studied in^32,36–39^. Zhi et al. improve the zero-shot pathology image analysis in^36^ by using crowd-sourced platforms such as medical twitter. The authors of the study^37^ present a foundation model for dermatological studies by pairing 100,000 images with natural descriptions in medical literature. The remaining unsupervised vision language pretraining methods optimize classification accuracy by improving the bidirectional contrastive objective^32^ or using multiple images and sections of the text report^38^.

Although saliency- or attention-based VL classification models can indicate the general region of interest, VL object detection frameworks enable more precise predictions, which is discussed next.

### VL object detection methods

VL information is used by dedicated object detection approaches, where newer approaches of VL detection are built on a DEtection TRansformer (DETR)^40^ framework by Carion, et al. . The method trains an additional transformer encoder-decoder network to determine the presence of a finding, which is based on the feature embeddings extracted by the image encoder backbone. In the medical domain, the application of DETR-based methods is typically based on numerous hypothesis boxes on high-resolution images (800-1024 pixels). The latest VL and DETR^40^ based detection methods such as GLIP^41^, OWL-ViT^42^ or Grounding DINO^43^ use image-text correlations, along with contrastive losses in transformer encoder-decoders or cross-attention mechanisms to improve detection performance.

In the above mentioned methods there is a trade-off between classification and localization that is exacerbated by domain misalignment^44^, requiring more annotations per class and processing of higher resolution images. Furthermore, the large number of evaluated hypothesis regions and class imbalance increases the average number of false positives (FP) detected, especially when high sensitivity in model performance is the goal. Consequently, training a robust medical abnormality detector is intricate, and requires the generation and evaluation of hundreds of hypothesis regions^45,46^. The performance gains of these VL detection systems are accomplished through pretraining over automatically annotated datasets, where textual information is linked to image regions. Here, the focus is often on enabling open-vocabulary or zero-shot detection performance by pretraining on previously seen text guidance data. This grounding pretraining information does not exist for the medical domain. Consequently, VL-based systems do not yet outperform dedicated abnormality detection systems.

Among these, one of the strongest contrastive object detection frameworks is Grounding DINO, whose performance gains primarily arise from its large feature extractor architecture and extensive supervised pretraining on approximately 1.8 million bounding box annotations. In contrast, our proposal Patch-CLIP builds upon unsupervised vision–language pretraining, enabling localization using only a lightweight fully connected layer appended to the frozen feature extractor. This design significantly reduces computational and data annotation requirements while retaining coarse localization capability making our proposal a scalable solution for CXRs.

Both VL classification and detection approaches provide valuable grounding capabilities; however, domain-specific medical abnormality detection systems consistently deliver higher accuracy and reliability, motivating their review in the following section.

### Vision-based abnormality detection methods

Open sourced and commercial abnormality detection systems employ a diverse array of methodologies to attain SOTA detection performance within the CXR domain. These systems typically use additional anatomical and contextual information extracted from clinical reports that improve upon straightforward detection algorithms. In one such study, Lian et al. exploit the structural relationships between anatomical and abnormality features using a Mask-RCNN framework, and an additional internal training dataset to achieve high detection performance^15^.

Similarly, Nguyen, et al.^46^, conducted a clinical validation of the performance of a commercial AI-based abnormality detector in 14 categories establishing a SOTA baseline on the VinDR-CXR dataset^3^. On the other hand, Ghesu et al.^5^ set the SOTA for abnormality classification and detection by pretraining on a dataset of 100 million images of different modalities using SwAV^40^ pretraining and finetuned on an internal dataset using FCOS technology^47^. This is a commercial product that is not open sourced and is our Reference Product Solution (RPS). In this study, we compare our results to both our RPS^5^ and baseline abnormality detection^46^ methods to maintain consistency with the metrics reported in the original dataset and the associated article.

## Method

The paper describes scientific research using retrospectively acquired anonymized data. The anonymization was performed in accordance with applicable laws and regulations before secure transfer was made to Siemens Healthineers for the study. Use of the data followed all the applicable license terms. The study does not involve any clinical or human subject research component to it. As such, IRB approval and informed consent are not applicable because there are no data privacy issues and no patients were impacted by the research (did not affect treatment or diagnosis).

The Patch-CLIP approach proposed in this manuscript is inspired by CLIP like pretraining to enable localization through a novel finetuning process. Specifically, the pretraining and finetuning scheme aligns the image patch embeddings and text embeddings in a shared feature space that is learned through a newly defined contrastive loss. The proposed approach is described in detail below.

### Linear combination loss for patch embeddings

Our pretraining framework introduces a novel downscaler network and a linear combination loss. The downscaler network consists of a series of convolution layers used as a pre-processing step. In our experiments, the downscaler has demonstrated better performance over conventional downsampling schemes such as bilinear, bicubic interpolations, etc. in reducing the image size to 224$\times$224 pixels^48,49^ (see ablation Table 7). To improve the compute efficiency, all input images are first uniformly downscaled to 900$\times$900 using bicubic interpolation and then further downscaled to the processing size using the convolution layers. The processing image resolution is fixed to leverage the CLIP pretrained weights. Identical pre-processing methodology is duplicated during the fine-tuning phase, as depicted in Fig. 1 in blue.

Next, we introduce a fully connected (FC) layer $\mathcal {F}$ that linearly combines the patch embedding outputs $\textbf{p}$ of the image encoder. Previous VL approaches^6^ have largely ignored these feature embeddings; however, our hypothesis is that these patch embeddings inherently encode the location information while enabling classification. The linearly combined embedding is then used to compute an additional loss. This linear combination loss denoted as $L_{lcl}$ allows the patch embeddings to encode more relevant spatial features that are derived from the text, thus allowing them to better align to the text embeddings after finetuning.

Conventional pooling operations (e.g., global average pooling or attention-weighted pooling) collapse patch features into a single vector before image–text comparison, which may overlook spatially discriminative regions. In contrast, $L_{lcl}$ explicitly supervises the combination of patch embeddings such that their learned weights form a semantically coherent representation aligned with the text embedding. This enables the network to adaptively emphasize the most relevant patches in a data-driven manner rather than relying on a fixed pooling prior^50^. Empirically, this formulation yields improved zero-shot classification performance (see Table 7).

To ensure the combination loss is computed in the common latent space, $\textbf{p} \in \mathbb {R}^d$ is first projected into the joint VL shared space, using the original projection layer $\mathcal {P}$, also shown in Fig. 1. This results in *n* embeddings $\mathbf {p'} \in \mathbb {R}^d$. We compute the cosine similarity of this combined embedding and text embedding to calculate the contrastive loss $L_{lcl}$. Mathematically, the pretraining loss is defined as

1 $$\begin{aligned} \begin{aligned} L_{pt\_total}&= \gamma _1 L_{global} + \gamma _2 L_{lcl}, \\ L_{global}&= CE(\mathbf {g'} \times \mathbf {t'}, Label), \\ L_{lcl}&= CE(\mathcal {F}(\textbf{p}_n') \times \mathbf {t'}, Label), \end{aligned} \end{aligned}$$

where $L_{global}$ is the original global CE loss, resulting from the correlation of the projected global embedding $\mathbf {g'}\in \mathbb {R}^d$ to the projected text embedding $\mathbf {t'}\in \mathbb {R}^d$, as seen in Eqn. 1. $\gamma _1, \gamma _2$ are weights for each loss component. $L_{lcl}$ is the loss from the correlation of the linear combination of the $n \in$ {1,*N*} patch embeddings $\mathcal {F}(\textbf{p}_n')$ with the text embedding, similar to $L_{global}$. *N* is the total number of patch embeddings and *Label* is the batch label that enables contrastive learning^2^.

### Patch embedding-based loss module for localization

We introduce the *Patch-based Loss Module* during finetuning, to enable localization. The patch-based loss module as shown in Fig. 1 has two components $L_{patch}$ and $L_{c\_patch}$, where the local patch features are aligned with the text labels so that the patch embeddings (output of the image encoder backbone) behave as local classifiers.

The complete finetuning loss, weighted by $\lambda _{\{1,2,3,4\}}$, is given by

2 $$\begin{aligned} L_{ft\_total} = \lambda _1 L_{global} + \lambda _2 L_{lcl} + \lambda _3 L_{patch} + \lambda _4 L_{c\_patch}, \end{aligned}$$

where $L_{global}$ and $L_{lcl}$ are as defined in the pretraining stage and the rest belong to the patch-based loss module. $\lambda _1, \lambda _2,\lambda _3, \lambda _4$ are the weights assigned to each loss term. The first component $L_{patch}$ aligns each individual patch embedding with the text embedding of the label “Finding” or “No Finding” and the second component $L_{c\_patch}$ aligns a combination of patch embeddings with the text embedding similarly. This combination is a learned representative image feature embedding, obtained from patch embeddings of interest, similar to attention-based pooling^51^. This is achieved by the *Learnable Combined Embedding* module $\mathcal {M}$ inside the *Patch-based Loss Module* in Fig. 1. We start with an explanation of how the patch embeddings are selected and then a detailed explanation of both the loss components.

#### Select patch embeddings

The patch embeddings of interest are marked by first converting the annotated bounding box coordinates to a patch *ground truth mask* $\textbf{G}$ which is based on the target model architecture. For e.g. a ViT-L/14 model processing a 224$\times$224-sized input image, yields a 16$\times$16 mask that indicates the presence (1) or absence (0) of the finding in each patch location. The mask indicates local patch-level labels that can be used later for loss computation. A patch is deemed to contain the finding if the finding occupies more than 10% of the patch area. $\textbf{G}$ is generated offline along with the relative sizes of the bounding boxes that is used during evaluation.

#### Patch loss $L_{patch}$

The original model produces image $\textbf{g}$ and text $\textbf{t}$ embeddings that are projected in a shared multi-modal feature space, resulting in $\mathbf {g'}$ and $\mathbf {t'}$. The global classification of the image is determined on the basis of the contrastive similarity in this shared feature space. In the first component of our proposed patch-based loss module, all of the patch-wise feature embeddings $\textbf{p}$ are also projected into the same shared feature space with a trainable *secondary projection layer* $\mathcal {S}$, resulting in $\mathbf {p''}$. This is depicted in the *Patch-based Loss Module* in Fig. 1 and necessitated by the disparity in summarizing global and local information. Next, the text encoder produces embeddings for both “No Finding” plus “Finding”, denoted as {$\textbf{bt}_0, \textbf{bt}_1$} where “Finding” is replaced by the class name of interest. The cosine similarity is computed between $\mathbf {p''}$ and either $\mathbf {bt_0''}$ or $\mathbf {bt_1''}$ based on the ground truth mask $\textbf{G}$ generated earlier, thus producing patchwise logits $p_{logits}$. A CE loss on all patch logits determines the final loss value to be back-propagated through the network, where the CE loss targets are also derived from $\textbf{G}$. Subsequently, the second projection layer $\mathcal {S}$ learns to locally classify each patch to the absence or presence of the finding, extracting local features that align towards or away from the text embeddings in the shared feature space. $L_{patch}$ is defined as

3 $$\begin{aligned} L_{patch}&= \sum _{n=1}^N CE(p_{logits,n}, \textbf{G}_n), \end{aligned}$$

4 $$\begin{aligned} p_{logits,n}&= {\left\{ \begin{array}{ll} \textbf{p}_n'' \times \textbf{bt}_0'' & \text {if } \textbf{G}_n = 0, \\ \textbf{p}_n'' \times \textbf{bt}_1'' & \text {if } \textbf{G}_n = 1. \end{array}\right. } \end{aligned}$$

$n \in \{1,N\}$, where *N* is the total number of patches, and CE indicates the cross entropy loss function with ground truth mask labels $\in$ {0,1} derived from $\textbf{G}_n$. $p_{logits,n}$ denotes the logit output of the $n^{th}$ projected embedding, indicating the cosine similarity of $\textbf{p}_n''$ to the relevant $\mathbf {bt''}$.

#### Combined patch loss $L_{c\_patch}$

In the second component of patch-based loss, we pass only the patch embeddings of interest. A subset of the total set of patch embeddings $\textbf{p}$ and positional embeddings $\textbf{pE}$ is chosen according to their location within the specified ground truth bounding box and is represented as $\mathbf {p_S}$ and $\mathbf {pE_S}$. These are processed by an attention pooling transformer $\mathcal {M}$, to learn a representative feature embedding. $\mathcal {M}$ is depicted in Fig. 1 as the *Learnable Combined Embedding* module.

Unlike the first patch loss, where each patch embedding is aligned independently with the corresponding text embedding, thereby effectively treating each patch as a local classifier, the combined patch loss aims to capture the relational and contextual structure of the finding. Specifically, the positional embeddings $\mathbf {pE_S}$ are incorporated to encode relative positional information between the patches that constitute the finding region. This allows $\mathcal {M}$ to learn not only the local appearance features within each patch but also their spatial configuration, leading to a more coherent and discriminative representation of the abnormality.

As before, this representational embedding is aligned to the text embedding of “Finding”. For images that do not contain any finding, a random patch is selected to align with the embedding of “No Finding”. This loss $L_{c\_patch}$ ensures that the combination of patches along with their location is learned by $\mathcal {M}$ to influence the class prediction. Therefore, $L_{c\_patch}$ is mathematically presented as

5 $$\begin{aligned} \begin{aligned} L_{c\_patch}&= CE(p_{c\_logit}, 1), \\ p_{c\_logit}&= \textbf{p}_{c}'' \times \textbf{bt}_1'', \\ \textbf{p}_{c}&= \mathcal {M}(\mathbf {p_S}, \mathbf {pE_S}), \\ \mathbf {p_S}&= \textbf{p}_n \cdot \mathbb {I}(\textbf{G}_n = 1), \\ \mathbf {pE_S}&= \textbf{pE}_n \cdot \mathbb {I}(\textbf{G}_n = 1), \end{aligned} \end{aligned}$$

where $\textbf{p}_{c}$ is the output of the Learnable Combined Embedding module $\mathcal {M}$ that learns a combined representation of $\mathbf {p_S}$ and $\mathbf {pE_S}$ corresponding to the location and characteristic of the “Finding”, as seen in Eqn. 5. $\mathbf {p_S}$ represents the subset of *n* patch embeddings that lie within the groundtruth region, which are selected according to binary mask $\textbf{G}$. $\textbf{pE}$ refers to the positional embedding associated with $\textbf{p}$, $\mathbf {pE_S}$ is the subset of position embeddings where the mask $\textbf{G}$ is 1, and $p_{c\_logit}$ is derived from the cosine similarity of the projected $\textbf{p}_{c}$ and $\textbf{bt}_1$, denoted as $\textbf{p}_{c}''$ and $\textbf{bt}_1''$. For further clarity, the pseudocode for the Patch-CLIP method is provided in the Appendix section A.

## Experimental results

We demonstrate the localization capability of our loss function on two datasets through the comparison of the Free-response Receiver Operating Characteristic (FROC) curves on various findings as well as their area-under-the-ROC curve (AUC) scores. The details of the datasets are discussed first, followed by the evaluation criteria and the results.

### Data preparation

The MIMIC-CXR^20^ pretraining dataset is a large and publicly available collection of 350,000 de-identified chest radiographs, of more than 65,000 patients along with their corresponding radiograph text reports. Each image is associated with rich clinical metadata, including patient demographics, radiographic findings, and relevant clinical notes. This dataset is used for pretraining the model. Curated by the MIMIC-III (Medical Information Mart for Intensive Care III) research group, this dataset is sourced from diverse healthcare settings and is widely used for training and evaluating machine learning models in medical image analysis.

A dataset of 212,000 de-identified DICOM images^8^ from an outpatient imaging center with multiple locations in Northeast United States is additionally used for pretraining. This dataset consists of consecutively acquired CXRs, between August 2020 and August 2022 from the archives, including both laterals and procedure reports. Retrospectively collected data was anonymized according to HIPAA guidelines at the source prior to secure transfer for the study. There was no intervention or any interaction with individual patients or impact on patient care. IRB assessment has determined the exempt status for the nature of the research project. The dataset includes images from multiple vendors and comprises patients 18 years or older. The associated CXR-reports from both datasets are minimally pre-processed to extract mainly the impressions and findings sections. Redundant sentences and filler words are removed to shorten the text length, and the remaining section of the report is ignored at the moment.

The VinDR-CXR dataset^3^ consisting of 18,000 anonymized images, manually annotated by a team of 17 experienced radiologists, is used for finetuning. The annotations include 22 local findings, delineating rectangles around abnormalities, and 6 global findings indicating suspected diseases at the case-level. The dataset is split into a training set of 15,000 scans, and 3,000 test scans. Based on the most common findings prevalent in both the pretraining and this dataset, as well as disregarding the rare findings class, we start with 8 findings for evaluation in this study. All tests are conducted using a binary classifier. Only one radiologist annotation per finding, selected at random is considered as the training file consists of multiple radiologist annotations for each finding in an image.

Additionally, a smaller internal CXR dataset of 16,953 anonymized DICOM images is used for further validation of this method^8^. The images include frontal view CXR images of patients with bounding boxes indicating the presence of 16 findings, provided by an expert panel of radiologists. We use two internal test datasets of 331 and 412 images for the binary classification of pleural effusion and pneumothorax.

### Experimental setup

In pretraining, a ViT-L/14 model is trained with 4 A100 GPUs of 40GB memory (batch size 10) and a ViT-B/32 model is trained on 4 HGX-1 GPUS with 32GB memory (batch size 64) using PyTorch, Distributed Data-Parallel (DDP), SGD optimizer (lr of 0.0001), and CE loss. The new FC weights are initialized with a Xavier uniform initialization with no bias. The model is pretrained using both MIMIC and the out-patient dataset of image-report pairs. The best model is selected based on the highest validation AUC after 4 epochs^6^ after 3 independent runs.

The patch embedding combining transformer is a simple 2 layer, 4 head transformer with an input size of 1024 and hidden size of 2048. A class token is concatenated to the sum of positional and patch tokens. The transformer returns the L2 normalized class token. Furthermore, to preserve pretrained weights, the FC layers and the combining transformer use an lr of 0.01, and the backbone uses an lr of 0.0001.

The finetuning is conducted on a single 20GB HGX-1 GPU node. Finetuning typically converges within 10 epochs. The global classification is calculated at 90% TPR^52^. A multi-GPU weighted distributed sampler addresses data imbalance during finetuning and images are normalized using dynamic scaling for the DICOM images^5^ without additional augmentations. Results are the average of 5 independent runs. The loss terms are weighted equally in our experiments.

### Evaluation criteria

**Fig. 2:**
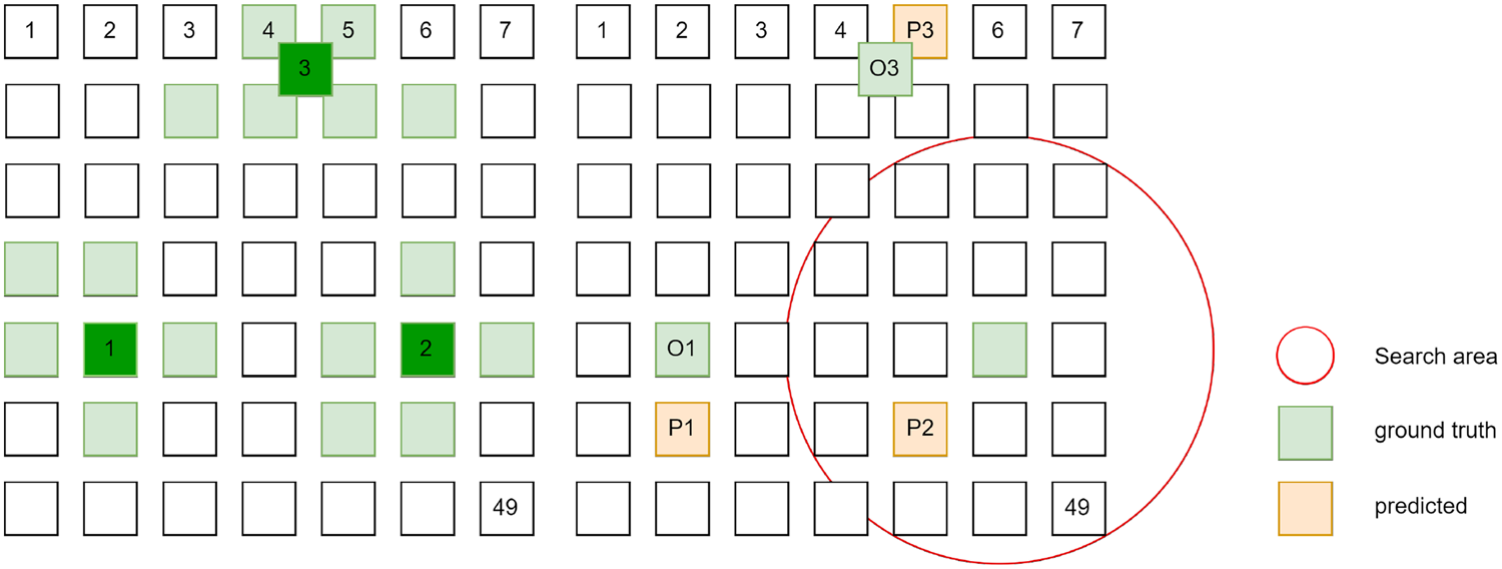
Patch based outputs denoting the evaluation criteria. ground truth mask (left) and the predicted patches (right), where the red circle denotes the search region within the allowed distance parameter.

Abnormality detection systems in medical image domain typically report the FROC curves^53^ instead of detection metrics like mean average precision (mAP) at 50% IoU^5,15,46,54,55,55–58^. The ambiguity inherent in defining precise boundaries in chest X-ray (CXR) abnormalities^15^, coupled with variability among observers, makes the instance-level FROC metric (also termed recall at fixed false positive rates) a practical choice for evaluation. In this scenario, the positive hit criteria is based on distance from the center of the predicted and ground truth regions, thus prioritizing the identification of the general region of the finding, crucial for guiding further diagnostic and treatment decisions.

Towards this end, we implement a hit criteria to determine the True Positive (TP) and False Positive (FP) in a test image. During evaluation, the patch embeddings are correlated with the embeddings of the “Finding” and “No Finding” text label. This generates patchwise logits indicating the probability of the finding’s presence, obtained through a softmax operation. As the threshold decreases, most patches show positive results, increasing the number of False Positives (FP). Hence, the FROC^53^ is plotted, depicting sensitivity vs. average FP. To further explain, the grid of patches is now treated as a low-resolution image, and we use the Hungarian matching algorithm which allows for correct matching between prediction blobs and ground truth boxes for multiple findings predicted in the grid. A hyper-parameter variable of allowed distance is scaled based on the relative ground truth box size to ensure a smaller search area for smaller findings. The search is conducted from the center of the ground truth box to the center of the predicted region. In our experiments, the allowed distance between predicted and ground truth is set to the minimum value of 1 patch to ensure the predicted regions lie within the ground truth region. The evaluation criteria are shown diagrammatically in Fig. 2 where the patches of the predicted finding must be within the red circle (allowed distance) shown on the right.

With this hit criterion, we now establish a patchwise and hence imagewise False Positive (FP), True Positive (TP) and False Negative (FN) for different thresholds and thus draw an FROC curve. Varying the threshold to achieve maximum sensitivity can result in an average FP greater than 1 per image. This is undesirable in a practical and useful clinical system, requiring that sensitivity at low average FP (determined by system design) be maximized.

### Comparison with SOTA

**Fig. 3:**
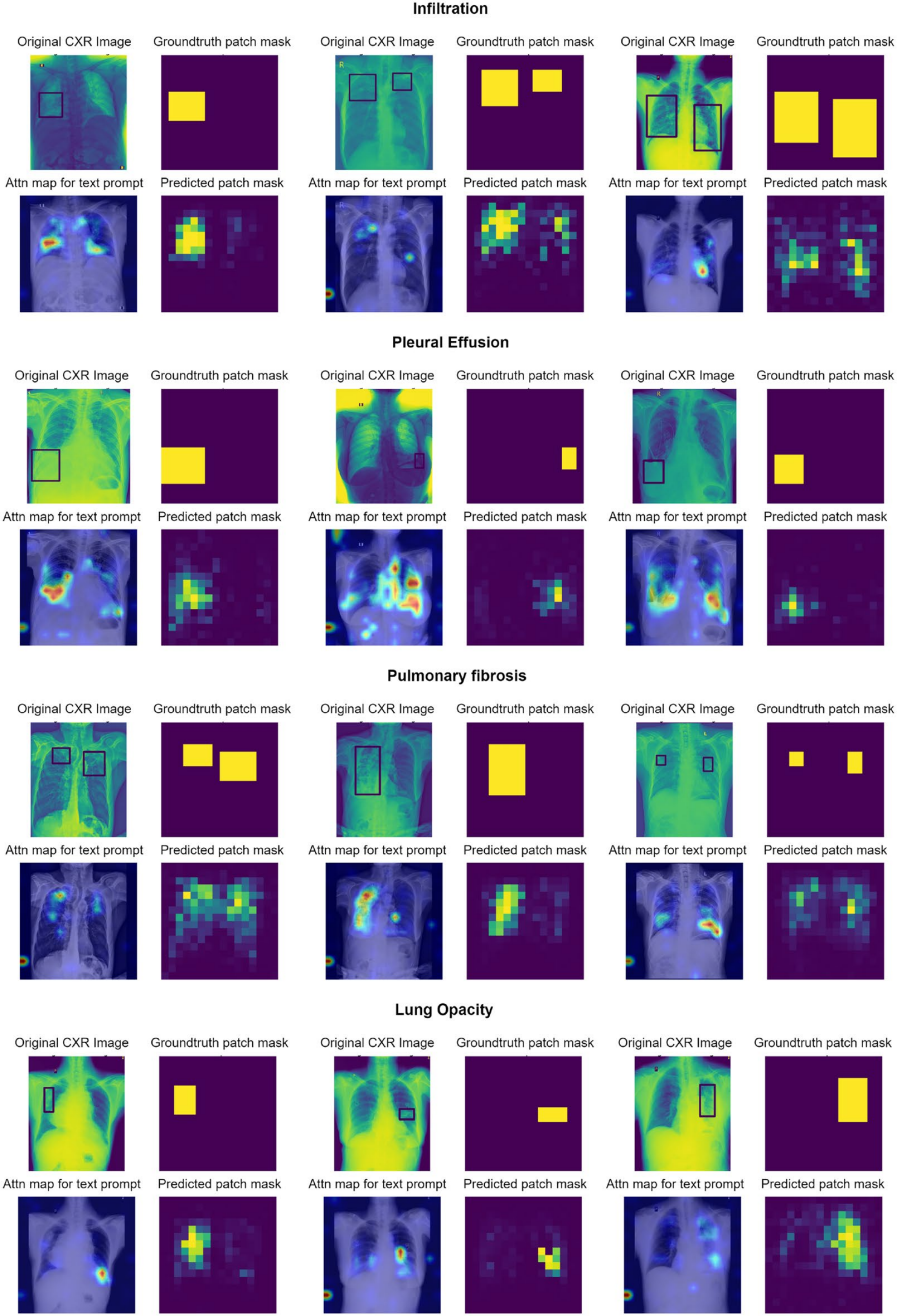
Example outputs for True Positive (TP) based on global predictions for infiltration, pleural effusion and lung opacity. The results are generated at 90% TPR^52^ for the global prediction and the un-thresholded prediction maps are generated at 0.2 FP for the patch probabilities. The top left sub-plot is the original CXR in DICOM format with bounding boxes, top right is the ground truth patch masks, bottom left is the attention map based on the text prompt and bottom right is the prediction map.

**Fig. 4:**
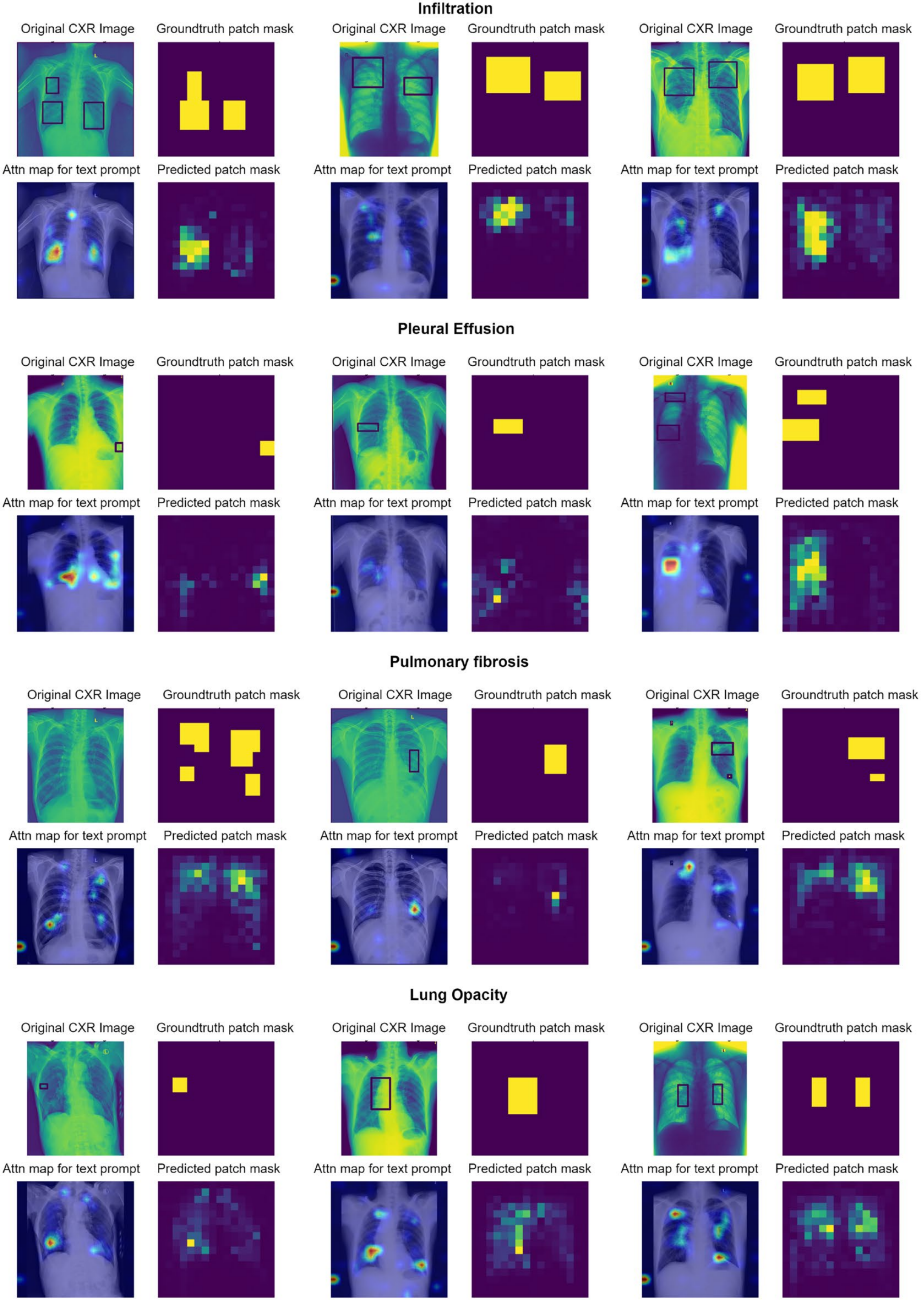
Instances of False Negative (FN) predictions with enhanced patch prediction maps in comparison to attention maps. The results are shown for infiltration, pleural effusion and lung opacity as examples at 90% TPR for the global prediction and 0.2 FP for the patch predictions.

In this section, the qualitative and quantitative results of localization on the VinDR-CXR dataset are presented. First, example patch prediction results are visualized as a group of 4 sub-figures each. Figures 3 and 4 show the patch prediction maps for multiple examples of four findings when the system makes a global prediction of True Positive (TP) and False Negative (FN) respectively.

The first top-left sub-figure indicates the original CXR including the bounding box annotation, and to the right is the ground truth mask generated from the bounding box coordinates. The second row of each figure displays the attention map response of Patch-CLIP to the text query eg. “Pleural effusion” and the last sub-figure on bottom right is the patch prediction output of the model. It is observed that the un-thresholded patch-wise logit scores (bottom right) often perform better compared to the attention map (bottom left) response of the model to some of the findings. The attention maps correlate to the number of positive samples in the pretraining dataset and difficulty of diagnosis, while revealing the areas where the model concentrates its attention to formulate the global prediction when prompted by the “Finding” text. The first row of figures are Patch-CLIP’s output when testing for “Infiltration”, the second for “Pleural effusion”, the third for “Pulmonary fibrosis” and the last for “Lung opacity”, with three examples for each finding.

**Fig. 5:**
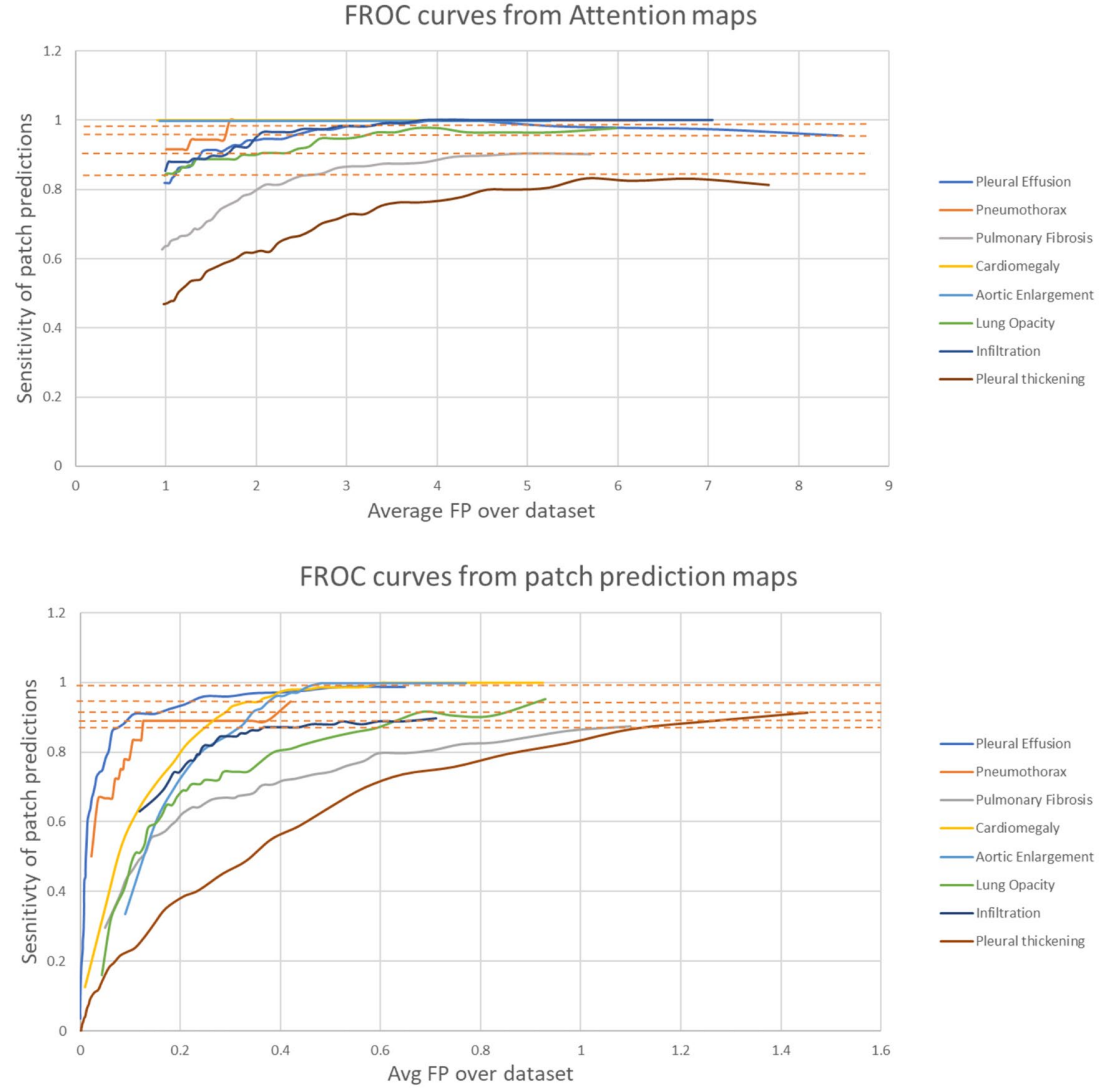
Compare the FROC curves generated from the attention maps and patch prediction maps for 8 findings from the VinDR-CXR dataset. For a fair comparison, the attention maps are treated similarly as the patch prediction maps and the detected regions are matched using the Hungarian algorithm. The x-axis in both figures are of different scale where the max FP from attention maps is higher.

It is observed that, when compared to the attention maps, the patch-wise logits provide a better indication of the location of the various findings. To quantify this improvement, Fig. 5 compares the FROC curves for eight findings that are calculated from the attention maps and the patch prediction maps, where it is seen that the sensitivities of the proposed system are higher at lower False Positive (FP) values. The FROC curve of the attention maps shows an overall higher average FP rate ranging from 1.0 to 8.0, while the patch prediction-based FROC curve typically has a maximum FP of less than 1. The higher number of average FP in the former is due to multiple spurious regions being highlighted irrespective of the actual finding location. The t-SNE visualization of the patch embeddings is illustrated in Appendix section B in Fig. S1. As expected, the sensitivities for findings such as cardiomegaly and pleural effusion are higher compared to findings such as pneumothorax or pleural thickening, due to the larger average size of the findings and lower spatial variance. The pretraining datasets also include a large number of positive samples for findings such as pleural effusion, resulting in Patch-CLIP having a better understanding of the visual features, as demonstrated by the overall better saliency maps.

Next, we compare the performance of Patch-CLIP against previous works on the VinDR-CXR dataset. Sensitivities at 0.25 FP and 0.5 FP are reported in Tables 1 and 2 alongside the results of a commercial SOTA EfficientNet abnormality detector system^46^ and grounding DINO^43^, a SOTA VL object detection model. At both operating points, our patch-based system performs better than Grounding DINO^43^, even though it processes images at much lower resolution (25%) and includes fewer additional layers. For the grounding DINO model, the top 300 predictions with scores greater than 0.005 are evaluated to compute the sensitivity numbers. Interestingly, compared to the SOTA abnormality detector^46^, patch-based pneumothorax sensitivities are significantly higher in our method due to the CLIP pretraining scheme, which shows higher AUC scores (97% vs. 84%). Though the MIMIC dataset has only 9,000 positive samples of pneumothorax, it has more than 30,000 negative samples with reports specifically mentioning the absence of pneumothorax. The CLIP method trains the model to recognize images without pneumothorax, improving the classification score on this test dataset which has only 18 instances of pneumothorax out of 3000 total test samples, thus effectively reducing the number of false positives. On the other hand, the abnormality detection system is a commercial product not available to the public and reports higher sensitivities at 0.25 FP for some findings, even with a stricter evaluation criteria of a fixed 40% Intersection-over-Union (IoU) of the predicted boxes. It is important to note that the model runs at a higher input resolution, while our method is sensitive to spatial resolution (patch size impacts performance). The grounding DINO model was evaluated at the default hyper-parameter settings, native higher resolution, and with a similar distance based evaluation criteria described previously.

**Table 1:** Comparison of average sensitivities at 0.25 FP for the commercial product SOTA abnormality detector^46^, a VL object detection model called Grounding DINO^43^ and the proposed Patch-CLIP. The abnormality detector results marked with a ^†^ are reported with an IoU of 40%, input resolution 1024$\times$1024 and the patch based detector is set to the allowed distance of 1 patch scaled with relative size of the finding (ensuring predicted boxes lie within the ground truth box regions), input resolution 224$\times$224. Both the Grounding DINO and our patch based system have similar distance based evaluation criteria for computing the FROC values. Data in parenthesis are 95% confidence intervals.

Category	abnormality detector^46^^†^	Grounding DINO^43^	$L_{patch}$	$L_{patch} + L_{c\_patch}$	$L_{patch} + L_{c\_patch}$
Image resolution	1024	800	224	224	224
Training data	100%	100%	100%	100%	10%
Cardiomegaly	0.965(0.94,0.98)	0.695(0.67,0.72)	0.834(0.78,0.90)	–	–
Lung Opacity	0.617(0.52,0.72)	0.402(0.35,0.46)	0.625(0.59,0.66)	0.701(0.69,0.72)	0.696(0.67,0.73)
Pneumothorax	0.639(0.47,0.80)	0.247(0.18,0.31)	0.850(0.82,0.88)	0.866(0.84,0.90)	0.789(0.75,0.83)
Pleural Effusion	0.898(0.84,0.95)	0.616(0.52,0.72)	0.860(0.83,0.91)	0.959(0.95,0.96)	0.925(0.92,0.93)
Aortic Enlargement	0.838(0.79,0.89)	0.560(0.55,0.57)	0.762(0.73,0.79)	-	–
In. Lung Disease	0.664(0.61,0.72)	0.354(0.34,0.37)	0.621(0.58,0.65)	0.719(0.71,0.73)	–
Infiltration	0.801(0.71,0.89)	0.635(0.58,0.69)	0.717(0.79,0.74)	0.783(0.78,0.79)	0.728(0.70,0.76)
Pulmonary fibrosis	0.568(0.51,0.62)	0.522(0.48,0.57)	0.498(0.45,0.55)	–	–
Pleural thickening	0.494(0.43,0.56)	0.522(0.51,0.53)	0.202(0.18,0.23)	0.488(0.47,0.50)	0.413(0.40,0.43)
Average	0.720(0.64,0.79)	0.506(0.46,0.55)	0.663(0.64,0.70)	0.753(0.74,0.77)	0.730(0.69,0.74)

**Table 2:** Comparison of average sensitivities at 0.5 FP for the commercial product abnormality detector^46^, a VL object detection model called Grounding DINO^43^ and the proposed Patch-CLIP. The abnormality detector results^†^ are reported with an IoU of 40%, input resolution 1024$\times$1024 and the patch based detector is set to the allowed distance of 1 patch scaled with relative size of the finding (ensuring predicted boxes lie within the ground truth box regions), input resolution 224$\times$224. Data in parenthesis are 95% confidence intervals.

Category	Abnormality detector^46^^†^	Grounding DINO^43^	$L_{patch}$	$L_{patch} + L_{c\_patch}$	$L_{patch} + L_{c\_patch}$
Image resolution	1024	800	224	224	224
Training data	100%	100%	100%	100%	10%
Cardiomegaly	0.965(0.94,0.98)	0.753(0.73,0.78)	0.976(0.96,0.99)	0.999(0.99,0.99)	-
Lung Opacity	0.756(0.66,0.85)	0.521(0.49,0.55)	0.703(0.68,0.73)	0.791(0.78,0.81)	0.773(0.75,0.80)
Pneumothorax	0.680(0.52,0.83)	0.299(0.22,0.38)	0.905(0.86,0.95)	0.928(0.91, 0.95)	0.911(0.88,0.94)
Pleural Effusion	0.927(0.88,0.97)	0.724(0.69,0.78)	0.896(0.89,0.90)	0.981(0.98,0.99)	0.945(0.93,0.96)
Aortic Enlargement	0.882(0.84,0.92)	0.685(0.66,0.71)	0.954(0.91,0.99)	0.998(0.99,0.99)	0.998(0.99,0.99)
In. Lung Disease	0.782(0.73,0.83)	0.470(0.45,0.49)	0.755(0.72,0.79)	0.761(0.75,0.78)	0.755(0.75,0.77)
Infiltration	0.861(0.79,0.93)	0.762(0.67,0.85)	0.858(0.84,0.87)	0.897(0.89,0.90)	0.871(0.85,0.89)
Pulmonary fibrosis	0.627(0.58,0.63)	0.619(0.59,0.65)	0.600(0.57,0.63)	0.754(0.75,0.76)	0.735(0.73,0.74)
Pleural thickening	0.608(0.54,0.68)	0.632(0.62,0.64)	0.312(0.27,0.36)	0.653(0.64,0.66)	0.547(0.53,0.56)
Average	0.788(0.72,0.85)	0.607(0.57,0.65)	0.774(0.75,0.80)	0.862(0.85,0.87)	0.817(0.80,0.83)

To further validate our proposed method, Table 3 and Table 4 presents sensitivities at 0.25, and 0.5 FP on the internal test datasets respectively, compared to our SOTA RPS^5^ processing 1024$\times$1024 resolution input images. The performance for pleural effusion is closer to RPS compared to pneumothorax as Patch-CLIP’s performance is notably influenced by the patch size and size of the findings. This is especially evident in pneumothorax, where sensitivity at 0.25 FP increases from 17% to 70%, when tested on a ViT-B/32 model and ViT-L/14 model. There is a marked performance difference of our system for pneumothorax on this dataset compared to the previous public dataset. This is in part due to the in-house system being especially optimized for pneumothorax and the VinDR system have a large class imbalance w.r.t. this finding with only 18 positive samples. Additionally, we also run the in-house RPS at a comparable 224$\times$224 resolution to demonstrate our method achieves higher sensitivities under similar operating and evaluation conditions.

**Table 3:** Comparison of average sensitivities at 0.25 FP with our RPS^5^ and Patch-CLIP for pleural effusion and pneumothorax test sets. Both systems count True Positive (TP) when the center of the predicted box is inside the ground-truth box. Data in parentheses are 95% confidence intervals where denoted.

Category	RPS^5^	RPS-224^5^	Patch-CLIP (Ours)
Pleural effusion	0.99	0.93	0.97(0.96,0.98)
Pneumothorax	0.97	0.43	0.70(0.68,0.72)
Average	0.98	0.68	0.83(0.82,0.85)

**Table 4:** Comparison of average sensitivities at 0.5 FP with our RPS^5^ and Patch-CLIP for pleural effusion and pneumothorax test sets. Both systems count True Positive (TP) when the center of the predicted box is inside the ground-truth box. Data in parentheses are 95% confidence intervals where denoted.

Category	RPS^5^	RPS-224^5^	Patch-CLIP (Ours)
Pleural effusion	0.99	0.95	0.99(0.98,0.99)
Pneumothorax	0.97	0.50	0.83(0.80,0.86)
Average	0.98	0.73	0.91(0.89,0.93)

Moreover, we evaluate the classification results in terms of AUC scores and compare them to Medical SOTA methods such as EfficientNet based abnormality detection system^27^, ResNet-50 based MedKLIP^9^, Swin-B based RTMDet^59^ and ResNet-50 based RetinaNet^14^. We also compare against VL models such as ResNet-50 based GLORIA^10^, ViT-L/16 based SLIP^33^ and the ViT-B/32 baseline CLIP^2^ under CheXZero^6^, ViT-B/32 based MERGE^39^, Swin-B based grounding DINO^43^ in Tables 5 and 6. Table 5 depicts the results on the public dataset and Table 6 depicts the results for the internal dataset. In Table 5 the classification results are competitive in most and outperform SOTA in key findings such as pneumothorax. The variance in performance can also be attributed to the differing image encoder backbones in each method. In addition, the SOTA RPS^5^ model achieves an AUC score of 98% for pneumothorax and 99% for pleural effusion.

**Table 5:** Comparison of AUC score for various findings in the VinDR-CXR dataset^3^ using various Medical and Vision-Language (VL) SOTA methods. The performance of our RPS is also included for pleural effusion and pneumothorax^5^. The standard deviation for all finetuning results are $< \pm 0.004$.

Category	Medical SOTA					Vision-Language SOTA					
	ViT	DualEncoder	RetinaNet	RTMDet	RPS	CheXzero	MedKLIP	SLIP	GLORIA	GDino	Patch-CLIP
	^27^	^27^	^14^	^59^	^5^	^6^	^9^	^33^	^10^	^43^	Ours
Cardiomegaly	0.96	**0.97**	0.95	0.93		0.96	0.94	0.92	0.32	0.90	**0.97**
Aortic Enlargement	0.96	**0.97**	0.88	0.80		0.91	0.86	0.86	0.90	0.76	0.93
Pleural Thickening	0.91	**0.92**	0.87	0.86		0.85	0.85	0.81	0.50	0.84	0.90
Pulmonary Fibrosis	0.91	**0.92**	0.90	0.90		0.84	0.94	0.77	0.28	0.86	0.90
Lung Opacity	0.90	**0.92**	0.83	0.85		0.88	0.84	0.83	0.89	0.80	0.90
Infiltration	0.90	0.91	0.91	0.88		0.88	0.83	0.79	0.90	0.90	**0.92**
Pleural Effusion	0.92	0.93	0.93	0.95	**0.99**	0.97	0.96	0.92	0.98	0.93	**0.99**
Pneumothorax	0.84	0.81	0.76	0.80	**0.98**	0.96	0.81	0.87	0.33	0.85	**0.98**
In. Lung Disease	**0.92**	**0.92**	0.72	0.81		0.87	0.84	0.79	0.83	0.79	0.90
Average	0.91	0.92	0.86	0.86		0.90	0.88	0.84	0.66	0.85	**0.93**

Additional comparisons in Table 6 include the fully supervised Imagenet pretrained ResNet-50 and the self-supervised (SSL) ResNet-50 SwAV^40^ pretrained model. In this context, the RPS system demonstrates superior performance compared to all other models. Meanwhile, Patch-CLIP achieves a comparable second place, notwithstanding the absence of pretraining on the 100 million image dataset.

**Table 6:** Comparison of classification AUC scores on the Internal Dataset with various Medical and Vision-Language SOTA models. Results are AUC classification scores for Pleural effusion and Pneumothorax. The standard deviation is $< \pm 0.004$.

Type	Model	Pleural effusion	Pneumothorax	Average
Med. SOTA	RPS^5^	0.994	0.977	0.985
	SSL SwAV^40^	0.979	0.933	0.956
	R-50^60^	0.984	0.921	0.953
	RetinaNet^14^	0.979	0.779	0.879
	RTMDet^59^	0.943	0.646	0.795
VL SOTA	Grounding DINO^43^	0.993	0.976	0.984
	Patch-CLIP (Ours)	0.991	0.932	0.961
	MERGE^39^	0.988	0.918	0.953
	ChexZero^6^	0.975	0.926	0.950
	MedKlip^9^	0.982	0.895	0.939
	SLIP^33^	0.925	0.918	0.922
	GLORIA^10^	0.836	0.723	0.779

### Ablation studies

In this section, we conduct a few ablation studies to understand the potential and limitations of our proposed model.

#### Impact of pretraining dataset size

**Fig. 6:**
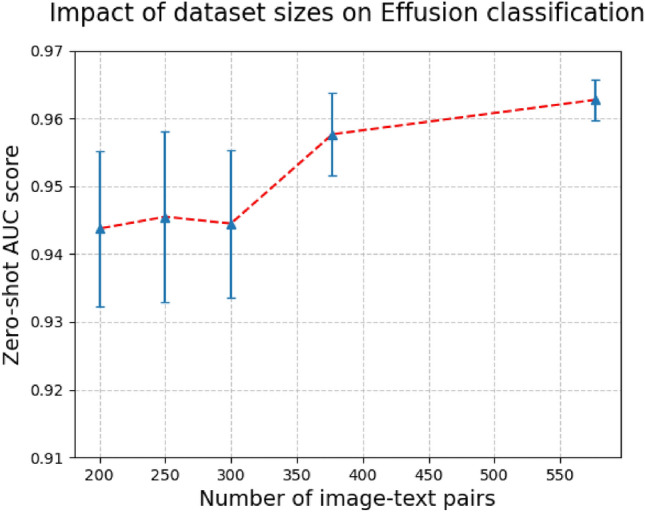
Increased zero-shot AUC score for pleural effusion, shown as the average of 5 independent pretraining runs using ViT-L/14 architecture for the image encoder.

Firstly, Fig. 6 shows the impact of the increased dataset on the zero-shot classification AUC score for pleural effusion, which in turn impacts the FROC scores. Pleural effusion has a relatively large number of positive samples (57,000) in MIMIC-CXR^20^, when compared to other findings such as pneumothorax (9000). Therefore, the improvement in zero-shot AUC score from the addition of the second dataset is higher for pneumothorax^61^, ranging approximately 8% compared to the performance improvement of 0.5% for pleural effusion. As the pretraining pretext task relies on a contrastive VL pairing within a batch, the zero-shot performance of the pneumothorax is less stable than pleural effusion. Performance is stabilized by using the second dataset and increasing the capacity of Patch-CLIP such as ViT-L/14.

#### Performance impact of proposed modifications

The Table 7 shows the impact of each of the components in the proposed module, when compared to the baseline CLIP method. The baseline implements the standard bicubic interpolation downscaler as implemented in torchvision and part of the data processing pipeline. We observe that the linear combination loss $L_{lcl}$ demonstrates a small improvement in the baseline zero-shot classification performance, also reflected in the finetuning results. The results are the average of 5 independent runs on a ViT-B/32 for pleural effusion. We select pleural effusion as it demonstrates relatively stable pretraining performance compared to other findings, due to the large number of positive samples available in the pretraining dataset. In addition, we compare the impact of including the linear combination of the patch embeddings vs. using standard pooling mechanisms like max pooling or average pooling.

**Table 7:** Ablation study of the various components of the proposed module on ViT-B/32 model for pleural effusion of the internal dataset. Results are average of 5 independent pretraining runs. The baseline implements the standard bicubic interpolation downscaler as implemented in torchvision.

Method	Image resolution	AUC
Baseline - CLIP with Bicubic interpolation downscaler	224	$0.949\pm 0.0086$
CLIP + with impression & findings in report	224	$0.953\pm 0.0069$
CLIP + learnable downscaler	1024	$0.957\pm 0.0045$
CLIP + learnable downscaler + $L_{lcl}$	1024	$\mathbf {0.961\pm 0.0035}$
CLIP + learnable downscaler + average pooling of patch embeddings	1024	$0.958\pm 0.0008$
CLIP + learnable downscaler + max pooling of patch embeddings	1024	$0.951\pm 0.0038$

#### Impact of spatial resolution and architecture

**Fig. 7:**
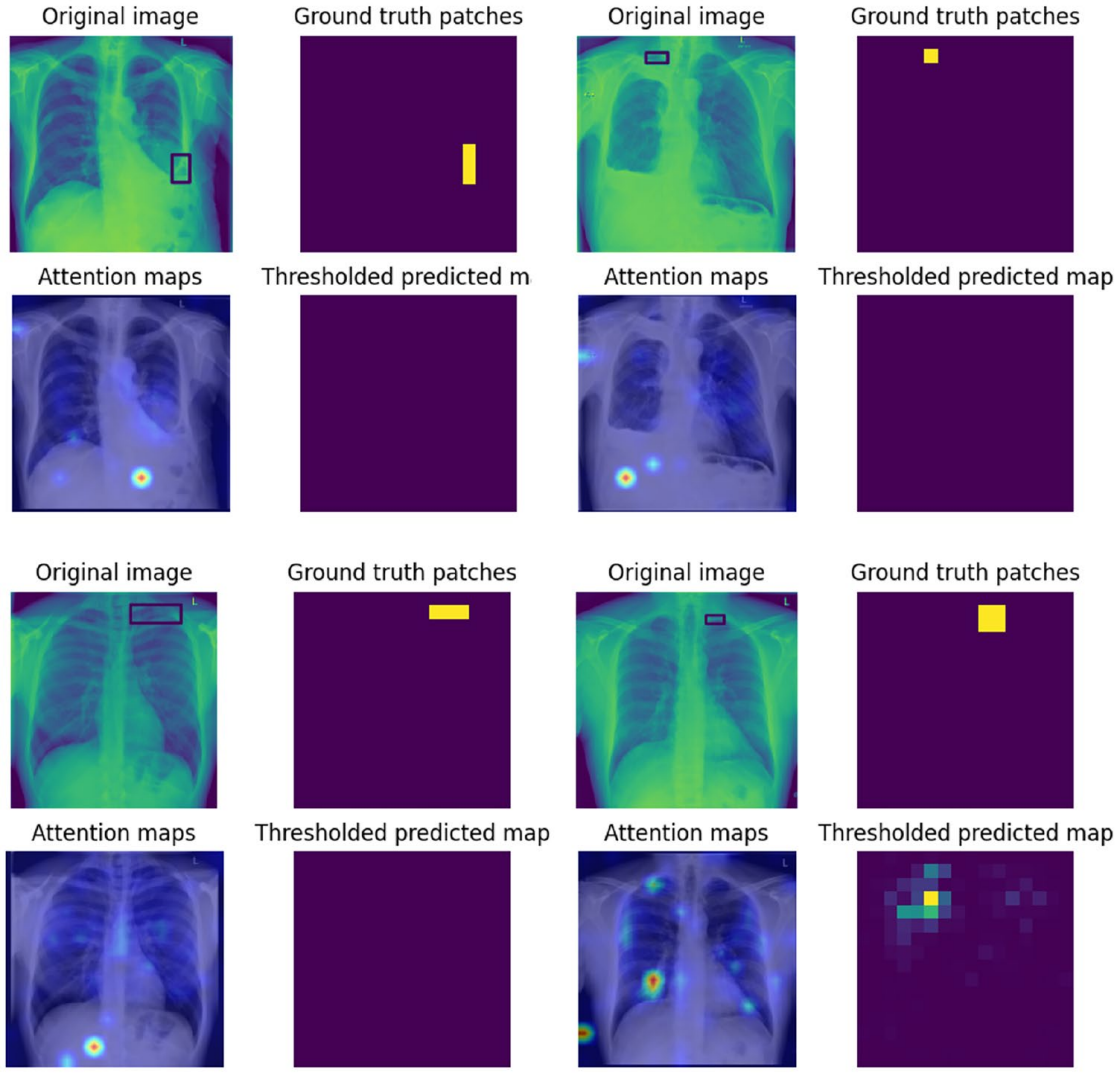
Examples of failure cases where the patch-prediction maps fail to predict the location of pneumothorax. The model also provides a global classification label of “No Finding” for these images.

Table 8 shows the improvement in sensitivity scores at 0.25 and 0.5 FP for pneumothorax between the ViT-B/32, ViT-B/16 and ViT-L/14 models. The improvements can be attributed to both the larger model capacity and the smaller patch size (32$\times$32 pixels vs. 14$\times$14 pixels). However, the model performance remains highly dependent on the patch resolution. Findings that are smaller than a patch, such as calcifications or smaller nodules, are often missed or poorly localized, indicating that the coarse patch-level representation limits the detection of fine-grained features smaller than the patch size. This is illustrated in Fig. 7 which indicates the failure of the patch-prediction maps for small-sized pneumothorax. In contrast, the model performs substantially better for findings that are spatially consistent or occupy larger regions, such as pleural effusion, devices or cardiomegaly. We expect that using even smaller patch sizes (e.g., 7$\times$7) or adopting more recent transformer variants that incorporate local attention mechanisms or hybrid CNN–ViT backbones could help overcome this limitation. While such architectures were not part of the initial investigation, they represent promising directions for future work.

**Table 8:** Impact of model architecture and patch size on sensitivity at 0.25 and 0.5 FP for pneumothorax on the internal dataset.

Model	Sensitivity @0.25 FP	Sensitivity @0.5 FP
ViT-B/32	$0.17\pm 0.6211$	$0.35\pm 0.3732$
ViT-B/16	$0.36\pm 0.3081$	$0.55\pm 0.0597$
ViT-L/14	$0.70\pm 0.2854$	$0.83\pm 0.0264$

## Discussion and conclusion

In this paper, we introduced a novel contrastive loss function designed to extract the implicit information from the positional encodings of the ViT image encoder in large scale VL models. The multi-task classifier leverages this inherent positional knowledge to tackle the detection objective by simultaneously constructing a local and global classifier.

Patch-CLIP combines patch contrastive losses and a linear combination loss, enforcing both local and global semantic alignment. Unlike prior patch- or attention-based approaches, Patch-CLIP achieves robust localization without requiring transformer decoders, region proposals, or dense annotations, making it computationally efficient and simple to deploy. Extensive evaluations on multiple CXR datasets demonstrate improved sensitivity at clinically relevant false-positive rates, high classification performance despite class imbalance, and strong generalization under data-scarce settings. Pretraining with a downscaler network and the linear combination loss further enhances baseline performance, and ablation studies confirm the complementary contributions of each component.

Leveraging large-scale self-supervised VL pretraining, our model demonstrates improved sensitivity results even when trained with only 10% of the dataset, leading to significant cost savings. Performance improves with larger dataset sizes and more positive samples of the rare findings, offering a scalable solution driven by the low cost of procuring VL data in Chest X-rays (CXRs). Our model achieves superior performance, despite processing lower resolution images, compared to the typical higher-resolution images used in object detection pipelines. Improvements in FROC scores between ViT-B/32, VIT-B/16 and ViT-L/14 indicate that smaller patch sizes in the image encoders can further enhance localization performance. However to note, is that the IoU could be relatively lower as the localization is discretized by the patch sizes. Furthermore, the model encounters challenges in accurately detecting findings that are smaller than a patch size, as seen during the evaluation of smaller findings.

In contrast, recent advancements in VL pretraining models, which are either patch-based or emphasize diverse pretraining methodologies, have been documented in various studies^32,38^. Such approaches can serve as supplementary baselines, enabling our method to be integrated atop these models to achieve further enhancements in performance. Consequently, our main comparative analysis is confined to our SOTA RPS and a leading VL model.

Future work will focus on developing a unified multi-class model with a more generalized open-vocabulary prompting mechanism and evaluation of this method on diverse medical datasets such as mammogram. Given that the CLIP model supports image-based queries, exploring few-shot localization for new findings presents an exciting avenue for further research. Although out of scope for the work presented here, we plan to evaluate our framework against more recent few-shot learning methods such as SGE^63^, DARA^64^, and other contemporary approaches^65,66^, to better contextualize Patch-CLIP’s medical few-shot and generalization capabilities within this evolving landscape. Conversely, while our current study focuses on static 2D radiology images that lack temporal information, extending Patch-CLIP to video-based medical imaging (e.g., ultrasound or fluoroscopy) represents an exciting direction for future research. In such settings, incorporating techniques from recent advances in video object detection, such as Feature Aggregated Queries for Transformer-based Video Object Detectors^67^ and DGRNet^68^ into Patch-CLIP could enable the model to leverage temporal consistency to further enhance detection stability and accuracy.

## Supplementary Information

Supplementary Information.

## Data Availability

The dataset utilized in this study comprised 2D X-ray imagery encompassing a total of 589,699 X-ray images, representing the chest region. The dataset was compiled from a combination of publicly available sources^20^ and proprietary, internal repositories^5^. Within this dataset, a subset of 16,693 chest X-ray images underwent meticulous annotation by a team of radiologists, encompassing the delineation of bounding boxes for pathologies spanning 16 distinct categories. For the purposes of this research, the 16,000 image subset was employed for the fine-tuning and assessment of the patch- CLIP technique. The study uses both data acquired from the resources that are publicly available^3^ as well as the data acquired for the project through collaboration and procurement agreements. The public data can be obtained using online resources and can be used under the applicable license guidance. Proper references are provided in the manuscript for the public data resources. The internal data acquired for the project can be available from the corresponding author upon a reasonable request.

## Code availability

The code used to generate the results in this study is publicly available at the Patch-CLIP GitHub repository hosted by Siemens Healthineers: https://github.com/Siemens-Healthineers/patch-clip. The repository is openly accessible to readers and includes the implementation together with setup, fine-tuning, and evaluation instructions. The software is distributed under the GNU General Public License v3.0 (GPL-3.0), and use of the code is subject to the terms of that license.
