# Supplementary material for: PatchCLIP enables region specific contrastive health record and image joint training with patch embedding loss
Source: Sci Rep. 2026 May 9;16:14688. doi: 10.1038/s41598-026-52235-x (PMC13157498; doi:10.1038/s41598-026-52235-x)
Supplement: Supplementary file 1 — Supplementary Information. [file 41598_2026_52235_MOESM1_ESM.pdf]

Appendix A Pseudocode of Patch-CLIP

Pseudocode for loss computation in the forward pass of Patch-CLIP training is provided below in Algorithm 1. The four loss terms are computed from the global class embedding, patch embeddings, and text embeddings generated from the image and text feature backbones. To maintain alignment in the representation, embeddings are projected through either the primary or secondary projection layers, and a CE loss is applied to all parts.

Algorithm 1: Patch-CLIP forward pass during finetuning

Input: Input image and text pair (I, T)
Output: Global loss $\mathcal{L}_{global}$, linear combination loss $\mathcal{L}_{lcl}$, patchwise loss $\mathcal{L}_{patch}$, and contextual combined patchwise loss $\mathcal{L}_{c\_patch}$

Step 1: Feature extraction
Extract global class embedding $\mathbf{g}$, text embedding $\mathbf{t}$, and N patch embeddings $\mathbf{p}$ from the feature backbone.

Step 2: Global contrastive loss
Project $\mathbf{g}$, $\mathbf{t}$ to $\mathbf{g}'$, $\mathbf{t}'$ and compute cross-entropy (CE) loss $\mathcal{L}_{global}$.

Step 3: Linear combination of patches
Pass all $\mathbf{p}$ through a fully connected (FC) layer $\mathcal{F}$ to generate a linear combination of patch embeddings, producing a single embedding.

Step 4: Local contrastive loss
Use this single embedding and $\mathbf{t}'$ to compute CE loss $\mathcal{L}_{lcl}$.

Step 5: Patchwise correlation
Pass all $\mathbf{p}$ through a secondary projection layer $\mathcal{S}$ to give $\mathbf{p}''$.

Step 6: Patchwise loss
For each projected embedding $\mathbf{p}''_{\mathbf{n}}$, correlate with "Finding" $\mathbf{bt}''_1$ or "No Finding" $\mathbf{bt}''_0$ based on the patchwise mask $\mathbf{G}$ and compute the CE loss L_{patch}.

Step 7: Ground-truth region selection
Select the subset of patch and position embeddings that lie within the ground-truth region denoted as $\mathbf{p_S}$ and $\mathbf{pE_S}$. Pass these to an attention pooling transformer $\mathbf{M}$. This generates a single representative embedding $\mathbf{p_c}$, which is projected to $\mathbf{p''_c}$ through $\mathcal{S}$.

Step 8: Contextualized combined patchwise loss
Compute a CE loss between $\mathbf{p}''_c$ and $\mathbf{bt}''_1$ denoted as $\mathcal{L}_{c\_patch}$.

return $\mathcal{L}_{global}$, $\mathcal{L}_{lcl}$, $\mathcal{L}_{patch}$, $\mathcal{L}_{c\_patch}$

Appendix B t-SNE plot of the patch-embeddings

Fig. S1 depicts the t-SNE of the patch embeddings for pleural effusion (top row) and pneumothorax (bottom row) with threshold of 0.2 and 0.5 to classify a TP, respectively. The embeddings are extracted for random 20 images from the internal proprietary test sets. For both findings, increasing the probability threshold reduces false positives while also decreasing true positives: for pleural effusion, TP drops from 216 to 190 and FP from 298 to 175; for pneumothorax, TP decreases from 60 to 25 and FP from 100 to 25, illustrating the trade-off between sensitivity and specificity.

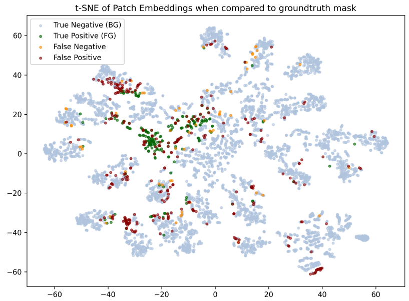

(a) t-SNE for pleural effusion at patch logit classification threshold of 0.2.

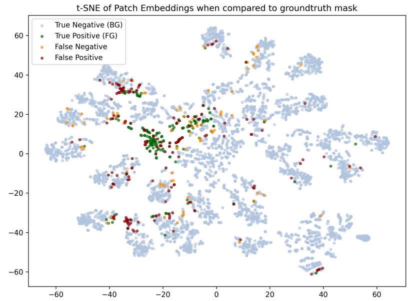

(b) t-SNE for pleural effusion at patch logit classification threshold of 0.5.

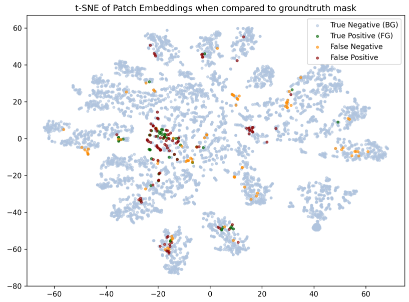

(c) t-SNE for pneumothorax at patch logit classification threshold of 0.2.

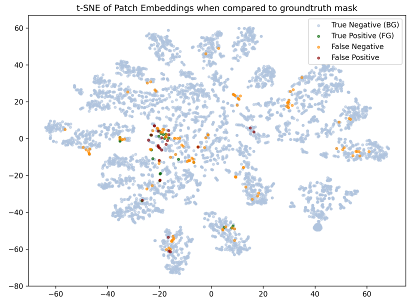

(d) t-SNE for pneumothorax at patch logit classification threshold of 0.5.

Figure S1: t-SNE plots showcasing the class separation between the foreground (FG) and background (BG) patches. The misclassified patches are also depicted.
